# The effects of crocetin, extracted from saffron, in chemotherapy against the incidence of multiple drug resistance phenotype

**DOI:** 10.22038/IJBMS.2018.29474.7118

**Published:** 2018-11

**Authors:** Navid Neyshaburinezhad, Maryam Hashemi, Mohammad Ramezani, Sepideh Arabzadeh, Javad Behravan, Fatemeh Kalalinia

**Affiliations:** 1 Biopharmaceutics and Pharmacokinetic Division, Department of Pharmaceutics, Faculty of Pharmacy, Tehran University of Medical Sciences, Tehran, Iran; 2 Nanotechnology Research Center, Pharmaceutical Technology Institute, Mashhad University of Medical Sciences, Mashhad, Iran; 3 Pharmaceutical Research Center, Pharmaceutical Technology Institute, Mashhad University of Medical Sciences, Mashhad, Iran; 4 Department of Pharmaceutical Biotechnology, School of Pharmacy, Mashhad University of Medical Sciences, Mashhad, Iran; 5 Biotechnology Research Center, Pharmaceutical Technology Institute, Mashhad University of Medical Sciences, Mashhad, Iran

**Keywords:** A2780, A2780-RCIS, Crocetin, MRP, Ovarian cancer cell line

## Abstract

**Objective(s):**

Crocetin, one of the main substances of saffron extract, has anti-cancer effects. Drug resistance proteins (e.g. MRP1 and MRP2) are important reasons for the failure of cancer therapy. We intended to investigate the efficacy of crocetin on MRP_1_ and MRP_2_ activity in human ovarian cisplatin-resistant carcinoma cell line (A2780-RCIS).

**Materials and Methods:**

The cytotoxic effect of crocetin was evaluated by the MTT assay. The efficacy of crocetin on MRP_1_ and MRP_2_ expression at mRNA level was studied by real-time RT-PCR. The effect of crocetin on the activity of MRP transporters was determined by drug efflux assay.

**Results:**

Crocetin decreased cell proliferation in the A2780 (IC_50_: 183±7 µM) and A2780-RCIS (IC_50_: 316±9 µM). Crocetin decreased the expression level of MRP1 (22±2 %) and MRP2 (48±8 %) in A2780-RCIS and inhibited MRP pumps function directly in A2780 (44±1 %) and A2780-RCIS (88±10 %) and indirectly in A2780 (32±2 %) and A2780-RCIS (48±15 %) respectively.

**Conclusion:**

Our findings showed that crocetin could quench drug resistance through modulation of MRP transporters in the drug resistant human ovarian cancer cells.

## Introduction

The enormous family of cancer diseases consist of abnormal cell growth correlated by the probability to attack or outspread to other sections of the body. In each year, cancers cause of about 9 million deaths that is about 16 % of human deaths worldwide (1). The major methods of cancer treatment include chemotherapy, radiation therapy, and surgery. Chemotherapy is khnown as one of the most effective cures for metastatic tumors. Chemotherapy is done as monotherapy or combination therapy, which has more beneficial effects in metastatic cases (2). Although chemotherapy is effective in early phases of cancer spreading, but in more advanced stages of cancer, cancer cells usually resist to chemotherapy agents. The simultaneous resistance of cancer cells to multiple drugs, without any structural or functional correlation (resistance to multiple drugs), is still one of the primary barriers for successful chemotherapy (3). The reason of cellular resistance to anticancer drugs are divided into two general categories including the factors that affect the access of cancer cells to anticancer agents, and genetic and epigenetic changes in cancer cells that affect their drug sensitivity. 

Multiple drug resistance (MDR) is one of the main reasons for unsuccessful cancer chemotherapy. Over-expression of the membrane transporter proteins cause generic MDR that ause low concentration of chemotherapeutic agents by throwing them out of cells (4). These pharmaceutical efflux proteins are part of the ATP-binding cassette transporters family that called ABC family (5). Due to its widespread distribution in the body, ABC proteins play an critical role in the transfer of various types of endogenous substrates. One of the major classes of the ABC family are MRPs (the multiple drug resistance associated proteins) that contains seven members (6). 

In the last decades, several researches introduced new herbal compounds with possible anti-neoplastic effects that has created a hope to expanding safer and more operative anti-cancer treatments (7). *Crocus sativus *(saffron) from *Iridaceae* family has been used as a food additive for years (8). Most of the therapeutic effects of saffron is due to safranal, picrocrocin and crocin (9). Crocin, as one of major carotenoids of saffron, has displayed anti tumoral effects both in *in vitro* and *in vivo*. Crocin could affect the action of cellular proteins, but the exact target of crocin and other carotenoids of the saffron have not been recognized yet (10). It has been previously showed that crocin has inhibition effect on MRP1 and MRP2 tranporters (11). Crocetin, a natural apocarotenoid dicarboxylic acid, is the central core of crocin (12). It showed cytotoxic effects in various cellular models and on different kinds of tumors including leukemia, ovarian and breast carcinoma, colon adenocarcinoma, liver, pancreas and lung cancer (13). In a study comparing the cytotoxic effects of crocin and crocetin, it has been shown that crocetin has 5 to 18 times more toxicity than crocin due to its structural difference, and the mechanism of toxicity is different too (14). So it is estimated that crocetin might possibly be used in the clinical interventions for prevention and treatment of cancer, in the near future. In this study, we intended to figure out the effects of crocetin on the MDR phenotype of human ovarian carcinoma cell lines (A2780 and A2780-RCIS) by evaluation the expression and function of MRP1 and MRP2 that are two important MRP transporters.

## Materials and Methods

***Materials***

Crocetin was extracted from saffron using the procedure represented in the Iran patent no. 84459. In order to determine the purity of crocetin, the crocetin absorption spectrum in dimethyl sulfoxide (DMSO) was detected in the ultraviolet light region at wavelengths of 200 to 700 nm. Two distinct peaks at wavelengths of 436 and 464 nm were found to be consistent with the pure crocetin absorption spectra.

Ovarian cancer cell lines (A2780 and its drug resistant A2780-RCIS derivative that overexpress MRPs) were kindly supplied by Professor Herman Lage (Molecular Pathology Department, Charite Campus Mitte, Berlin, Germany). In order to preserve the cisplatin-resistant phenotype of A2780-RCIS cells, the culture medium was supplemented with cisplatin 33.3 µM and replaced with cisplatin-free medium, one week before each experiment. 

RPMI-1640 and fetal bovine serum (FBS) were obtained from Gibco (USA) and Biosera (UK), respectively. Doxorubicin (Adriamycin), penicillin-streptomycin and MTT were purchased from Sigma-Aldrich, Germany. DMSO and trypan blue were obtained from Roche Applied Science, Germany. Other chemicals and solvent were achieved from Merck (Darmstadt, Germany). RNA extraction kit and cDNA synthesis kit were purchased from Parstoos, Iran and Real-time EXPRESS One-Step SYBR GreenER™ Kit was obtained from Parstoos, Iran.

**Figure 1:**
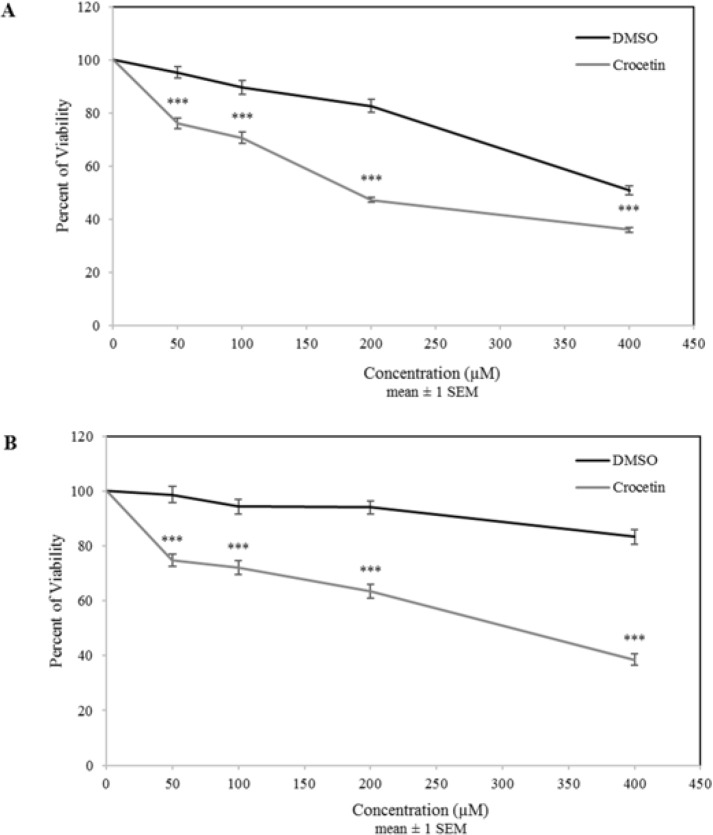
The effects of crocetin on cell viability of A2780 (A) and A2780-RCIS (B) cell lines. The cells were incubated with different concentrations of crocetin (0-400 µM) at 37 ^°^C for 48 hr. Cell viability was evaluated by the MTT assay and compared with control. Each experiment was repeated independently three times in triplicate tests and data are shown as mean ± SEM. ***P≤ 0.001

**Figure 2:**
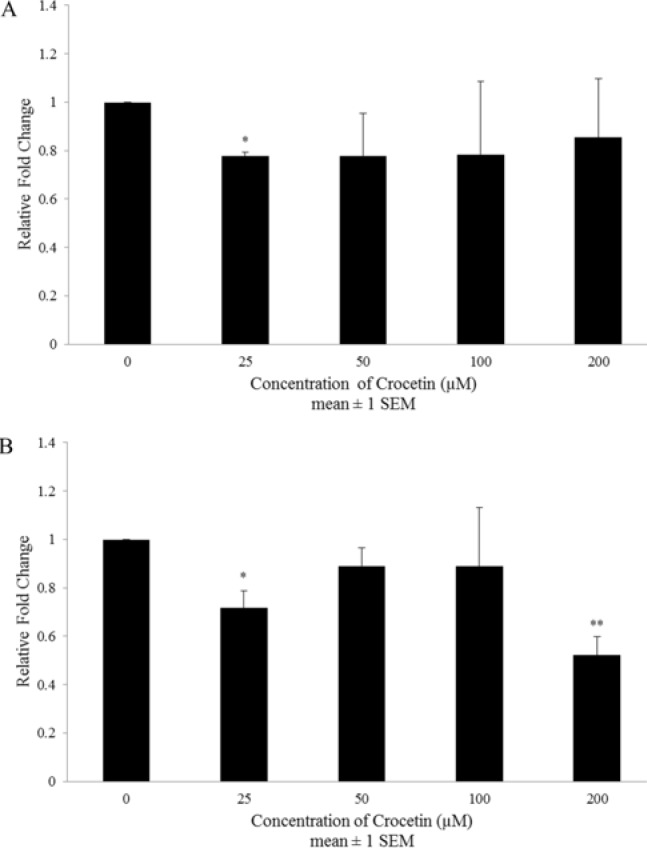
The effects of crocetin on MPR1 (A) and MPR2 (B) mRNA expression in the A2780-RCIS cell line. The cells were incubated with different concentrations of crocetin (0-200 µM) at 37 ^°^C for 48 hr. Effects of crocetin on MRP1 and MRP2 mRNA were evaluated by the Real-time RT-PCR. Each experiment was repeated independently three times and data are shown as mean±SEM. *P≤ 0.05; **P≤ 0.01 vs. control

**Figure 3:**
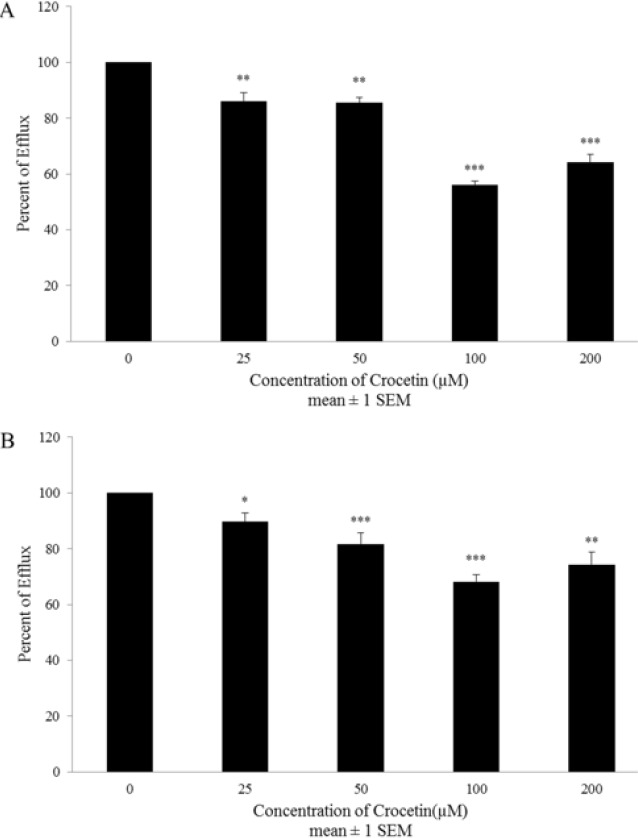
The direct (A) and indirect (B) effects of crocetin on the function of MRP transporters in A2780 cell line. The cells were incubated with different concentrations of crocetin (0-200 µM) at 37 °C for 1 to 48 hr. Inhibition effect was evaluated by the drug efflux assay. Each experiment was repeated independently three times in triplicate tests and data are shown as mean±SEM. *P≤0.05; **P≤0.01; ***P≤ 0.001 vs. control

**Figure 4:**
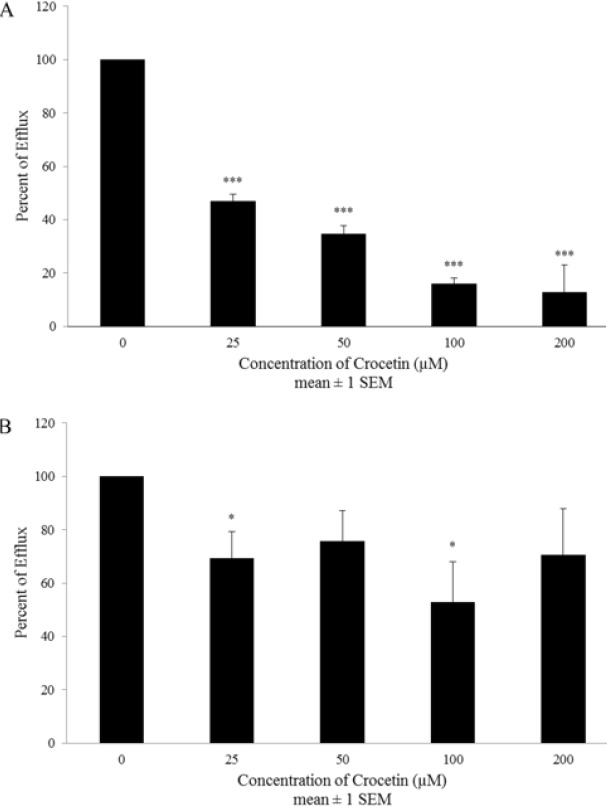
The direct (A) and indirect (B) effects of crocetin on the function of MRP transporters in A2780-RCIS cell line. The cells were incubated with various concentrations of crocetin (0-200 µM) at 37^ °^C for 1 to 48 hr. Inhibition effect was evaluated by the drug efflux assay. Each experiment was repeated independently three times in triplicate tests and data are shown as mean±SEM. *P≤0.05; ***P≤0.001

***Preparation of the crocetin solution***

For each experiment, crocetin was dissolved in DMSO to a final concentration of 60 mM and kept at −20 ^°^C. Crocetin was diluted by complete culture medium to its final concentration (25, 50, 100, 200 and 400 μM), exactly before each experiment. The maximum concentration of DMSO in 400 μM crocetin solution was about 0.65 % (v/v) while the safest range of DMSO is up to 0.5% (v/v) (15). Therefore, the cytotoxic effects of DMSO on tested cell lines were evaluated by MTT assay. 

***Cell culture***

Cells were routinely cultured in RPMI-1640 contained FBS and penicillin/streptomycin and stored at 37 ^°^C in CO_2_ 5%. This study was approved in the Research Ethics Committee of Mashhad University of Medical Sciences, Mashhad, Iran. 

***MTT cytotoxicity assay***

The cytotoxic effects of crocetin on the tested cell lines, were determined by MTT cytotoxicity assay. Ten thousand cells were seeded in 96-well plates and treated with various concentrations of crocetin (0-400 µM). The control wells had DMSO at equivalent concentrations to those used for the test mixtures. Cell viability was evaluated after 48 hr by adding MTT in PBS solution (0.5 mg/ml) to each well for 4 hr. Finally, the crystals of formazan were dissolved in 200 µl of DMSO and absorbance was assessed by an ELISA plate reader (BioTek, Germany) at 550 and 630 nm (test and reference wavelength, respectively).The ratio of OD_test_/OD_control_ × 100 was reported as the percentage of cell viability and the IC_50_ values were intended as the concentration of crocetin that decreased the cell viability to half.

***Real-Time RT-PCR***

The MRP_1_ and MRP_2_ mRNA expression of drug resistant cells (A2780-RCIS) was evaluated by real-time RT-PCR after treating cells with various concentrations of crocetin (0-200 µM) for 48 hr. Total cellular RNA of 5 × 10^5 ^cells was extracted by Parstoos RNA isolation kit. The total RNA concentration and the quality of them was measured by a NanoDrop 1000 spectrophotometer (Thermo Fisher Scientific, USA) and the acceptable range A_260_/A_230_ and A_260_/A_280_ ratios was among 1.8 to 2.2. cDNA was synthesized using Parstoos cDNA kit using 300 ng of total RNA. Real-time RT-PCR tests were performed by the Parstoos SYBR green master mix Kit and real-time cycler step-one PLUS ABI (ABI, USA). The sequences of primers were: MRP_1_: 5’-GTGTTTCTGGTCAGCCCAACT-3’ (forward) and 5’-TTGGATCTCAGGATGGCTAGG-3’ (reverse) (11); MRP_2_, 5’-AGCAGCCATAGAGCTGGCCCTT-3’ (forward) and 5’-AGCAAAACCAGGAGCCATGTGCC-3’ (reverse) (16); β-actin: 5’-TCATGAAGTGTGACGTGGACATC-3’ (forward) and 5’-CAGGAGGAGCAATGATCTTGATCT-3’ (reverse) (17). Reactions were carried out with an initial activation step at 94 ^°^C for 15 min followed by 40 cycles at 95 ^°^C for 15 sec and 60 ^°^C for 1 min as PCR amplification cycles. The specificity of primers was evaluated by melting curve analysis. The expression levels of MRP_1_ or MRP_2_ were normalized to the β-actin by the step-one PLUS system. The comparative expression level of tested genes were informed as the fpllowing equation: (target/reference ratio of the treated cells)/ (target/reference ratio of the untreated control cells) (11). 

***Efflux assay***

Doxorubicin and furosemide are known to be MRP_2_ substrate and MRP pump inhibitor, respectively (18, 19). In this study we aimed to find out whether crocetin can inhibit the efflux of doxorubicin from the A_2780_ and A2780-RCIS cells. The direct and indirect effects of crocetin on the inhibition of function of MRP transporters were evaluated by efflux assay. For evaluation of direct intraction of crocetin with MRP transporters, A2780 cell lines were exposed to crocetin (0-200 μM) and 10 μM doxorubicin in the presence or absence of furosemide (1 mM) (19) for 1 hr. After that, the medium was shift to the fresh culture medium in the presence or absence of furosemide (1 mM) for 3 hr. Then the supernatants were transferred to the black 96-well plates and doxorubicin content was assayed spectrofluorimetrically at 470 and 585 nm (excitation and emission wavelength, respectively). For evaluation the indirect effects of crocetin on the function of MRP transporters (by changing their expression), all cells were exposed to crocetin (0-200 μM) for 48 hr. Then, the supernatants were replaced with 10 μM doxorubicin in the presence or absence of furosemide (1 mM) for 1 hr. Finally, the supernatants were replaced with fresh culture medium with or without furosemide (1 mM) for 3 hr and continued protocol as described in the previous section. Performance level of drug resistance transporter was evaluated by percent of doxorubicin efflux by using the following ratio: ((Fluorescence amount in the absence furosemide in the sample-Fluorescence amount in the presence of furosemide in the sample)÷(Fluorescence amount in the absence of furosemide in the control sample-Fluorescence amount in the presence of furosemide in the control sample))× 100 (20).

***Statistical analysis***

Data (mean±SEM) were reported from three independent experiments. Statistical analyses were performed by SPSS version 16.0 using ANOVA, with the Tukey’s *post-hoc* test. A value of *P*<0.05 was considered statistically significant.

## Results

***Cytotoxicity assay***

To examine the effects of crocetin on the ovarian cancer cells viability, all cells were incubated with various concentrations of crocetin (0–400 μM) for 48 hr and then analyzed by MTT cytotoxicity assay. The results showed that crocetin had a concentration-dependent suppresive effects on the proliferation rate of the A2780 cells (Figure 1a). Crocetin displayed comparable inhibitory effects with lesser potency in A2780-RCIS cells (Figure 1b). IC_50_ values of crocetin for A2780 cell and its drug resistant derivative, A2780-RCIS cell, were determined 183±7 µ M and 316±9 µ M, respectively (*P< *0.05 vs. control).

***The effect of crocetin on the gene expression of MRP***_1_ ***and MRP***_2_

In order to assess the effects of crocetin on the expression of MRP_1_ and MRP_2_ at mRNA level, the resistant cells (A2780-RCIS) were treated with various concentrations of crocetin (0–200 μM) for 48 hr. Real-time RT-PCR results displayed that crocetin can reduce the expression levels of MRP mRNA in a concentration-independent manner. Maximum inhibition was statistically significant at 25 µM for MRP_1_ mRNA (up to 22 ± 2%) (Figure 2a) and 200 µ M for MRP_2_ mRNA (up to 48 ± 8%) (Figure 2b) in A2780-RCIS cells.

***Effect of crocetin on MRP***_1_ ***and MRP***_2_ ***efflux activity***

Exposure of A2780 cell line to all concentrations (0-200 µM) of crocetin decreased doxorubicin efflux in both direct (up to 44±1 % at 100 µM) (Figure 3a) and indirect (up to 32±2 % at 100 µM) (Figure 3b) efflux assay. Interestingly in A2780-RCIS cell line, direct suppression of MRP transporters was statistically signifcant in all concentrations (0-200 µM) (Figure 4a) and maximum inhibition (88±10 %) being reached at 200 µM. the results of indirect inhibition of MRP transporters by crocetin showed statistically significant maximum inhibition at 100 µM (48±15 %) (Figure 4b). Totally it has been shown that direct inhibition was more efficient than indirect inhibition.

## Discussion

Cancer is still one of the most challenging diseases. Chemotherapy is the most common way to treat cancer, but its effectiveness can be reduced by multi-drug resistance (MDR). One of the major mechanisms in MDR is the presence of drug transporters that pump chemotherapeutic agents out of the cell (21). Crocetin is a derivative of crocin, one of the major components of saffron extract. Recent studies have shown that crocetin exhibits many anti-cancer properties, including suppression of nucleic acid synthesis and induction of apoptosis (22). In this study, we assessed the effects of crocetin on the function and expression of MRP_1_ and MRP_2_ transporters in the human ovarian carcinoma cell line. 

It has been formerly displayed that MRP_2_-over-expression in human cancer cell lines raise the resistance to doxorubicin (23), while inhibition of MRP_2_ expression improve cell sensitivity to doxorubicin (24). According to these studies, doxorubicin has been considered as a substrate of MRP_1_ and MRP_2_ transporters. In our previous study, it has been shown that the expression level of MRP_1_ and MRP_2_ in the A2780-RCIS cell line was 1.29 and 13 times more than their expression level in parental sensitive A2780 cell line (11). Also, it has shown that doxorubicin decreased the A_2780_ cell viability with more severity in compare with the A2780-RCIS cells. These findings proposed that the more expression level of MRP transporters in A_2780_-RCIS cause more efflux of doxorubicin that caused its lack of intracellular concentration and lower cytotoxic activity in this drug-resistant cell line (11). Interestingly, crocetin has shown a comparable inhibitory schema on cell growth of tested ovarian cancer cells in which its anti-proliferation activity had less potency in A_2780_-RCIS in comparison to the A_2780_ cell line. These findings propose that crocetin might be a substrate for MRP transporters. 

The results of cytotoxicity assay showed that DMSO had more cytotoxicity in A_2780_ parent cells than the resistant cells (A_2780_-RCIS). It was consumed that the reason of this phenomena is the finite presence of MRP1 and MRP2 pumps in parent cells in comparison to resistant cells. Although the cytotoxicity of DMSO is high in parent cells but the effect of crocetin compared to DMSO is statistically significant. Also, in order to prevention of undesired effect of DMSO on the results of this study, lower concentration than 200 µM have been used for other tests.

In this research, we proposed to assess the effects of crocetin on MRP transporters expression and function. The MRP activity evaluation has been performed in two ways, short term disposal with crocetin to check the direct interaction between crocetin and existing active MRP transporters, and long term disposal with crocetin to check the indirect effect of crocetin on the activity of MRP transporters as a result of its regulations on their expression level. Interestingly, crocetin could decrease doxorubicin efflux after 1 and 48 hr in both drug-resistant A2780-RCIS cell line and A2780 parent cell. But the direct inhibition effect was more efficient than indirect inhibition effect in both resistant and sensitive cells. Then again, the real-time RT-PCR findings displayed that crocetin meaningfully decreased the MRP_1_ and MRP_2_ mRNA expression level at only two concentrations (25 µM for MRP_1_ and 25 and 200 µM for MRP_2_) in A2780-RCIS cells. Our results showed that the main path of MRP transporters inhibition by crocetin is directly by inhibition of efflux activity of these two transporters and the reduction of MRP gene expression may not be the major path for increasing doxorubicin cytotoxic effects on cisplatin resistant ovarian carcinoma cells. 

It has been previously showed that some carotenoids are competitive inhibitors of ABC-transporters (25, 26). They (crocin, β-carotene, canthaxanthin, retinoic acid, and fucoxanthin) can utilize MDR reversal and increase the cytotoxic effect of chemotherapeutic agents in human CEM/ADR5000 and Caco-2 cells (27). Also it has been showed that crocetin induces cytotoxic effect and increases vincristine-induced cancer cell death in MCF-7/VCR (the vincristine-resistant breast cancer cell line) and it can be used as a chemosensitizer for vincristine (28). Entirely, these findings proposed that simultaneous use of crocetin with chemotherapeutic agents in cancer treatment might be an effecient method to increase the effectiveness of chemotherapy and modulate the influence of MDR.

## Conclusion

In this study we intended to figure out the capability of crocetin on suppressing MDR in human ovarian cancer cells by inhibiting MRP_1_ and MRP_2_ transporters. The outcomes showed that crocetin could reduce the MRP efflux function and perform as MDR reversal agent that increase the cytotoxicity effects of doxorubicin in the A2780-RCIS. This study proposed that the usage of crocetin in combination with chemotherapeutic drugs in cancer therapy could be an effective way to increase the efficacy of chemotherapeutics and limiting the impact of MDR transporters.
